# A gastrointestinal stromal tumour in the distal ileum—a rare presentation in Saudia Arabia: case report

**DOI:** 10.3389/fsurg.2026.1787138

**Published:** 2026-04-23

**Authors:** Mohammad Shawir, Mohammad Abdulkarim, Mohammed Elnibras, Haneen Brnawi, Marei Al amari, Roaa Ghazi Khan, Elsadig Shiekedien, Yazeed Al Jabri

**Affiliations:** 1 Department of Surgery, Faculty of Medicine, University of Tabuk, Tabuk, Saudi Arabia; 2 Department of Surgery, King Khalid Hospital, Ministry of Health, Tabuk, Saudi Arabia; 3 Department of Obstetrics & Gynaecology, Faculty of Medicine, University of Tabuk, Tabuk, Saudi Arabia

**Keywords:** Cajal's cells, GIST, imatinib, KIT, surgery, treatment, tumour

## Abstract

**Introduction:**

Gastrointestinal stromal tumours (GIST) are an extremely rare case in Saudi Arabia. These tumours arise from the smooth muscle cells of Cajal in the interstitium, a key component of Gastrointestinal tract (GIT) mesenchymal tissue.

**Case report:**

A 31-year-old male arrived at our emergency department with central abdominal pain, nausea, vomiting, and absolute constipation persisting for four days. Upon examination, he exhibited hypotension, tachycardia, and tenderness in the central abdomen. The provisional working diagnosis based on the abdomen computed tomogram (CT) indicated a pelvic tumour to the right of the lower midline. Contrast CT suggested a distal ileum inflammatory mass vs. a tumour. Operative results verified a tumour at the distal ileum accompanied by a dilated, obstructed volvulus in the small bowel. A histoinmunohistochemical analysis showed the presence of a gastrointestinal stromal tumour (GIST), demonstrating diffuse positivity for CD117 (Ckit), focal positivity for CD34, and negative results for S100 and desmin.

**Conclusion:**

It is critical to avoid tumour rupture during surgical resection, as this may result in tumour implantation or recurrence. Because the tumour was fragile and fragmented during dissection, a positive outcome may not be possible; consequently, our case requires strict surveillance and CT follow-up. The operating surgeon and histopathologist must agree on labelling specimens before sending them to the lab. Furthermore, research must investigate epidemiological issues, therapeutic methods, and follow-up procedures in Saudi Arabia. Guidelines for managing and following up on these cases should be developed based on the agreed-upon processes.

## Introduction

Gastrointestinal stromal tumours (GIST) are well known as tumours arising from the smooth muscle cells of the interstitial cells of Cajal, an essential component of the mesenchymal tissue of the gastrointestinal system (GIT) (1). (GISTs) are predominantly found in the stomach (60%), small intestine (40%), colon and rectum (5%), and oesophagus (5%), respectively. They are infrequently found in the mesentery, retroperitoneum, omentum, liver, gallbladder, pancreas, and urinary bladder, referred to as extra gastrointestinal stromal tumours (EGISTs), where distant metastases most commonly occur.

Each year, the United States reports approximately 5,000 cases of GIST, primarily affecting individuals aged 50 and 70. The incidence of GIST is evenly distributed between males and females. (GISTs) represent approximately 1%–3% of all gastrointestinal tumours and are recognised as the most prevalent mesenchymal neoplasms (2, 3). The prevalence in Saudi Arabia remains undetermined due to the absence of an official governmental data registry up to this writing (4). From 1997 to 2018, Saudi Arabia reported a total of 204 cases of GIST, distributed by geographic location: 6 cases in the southern region (4), 37 cases in the western region (5), 36 cases in the eastern region (6), and 125 cases in the central Riyadh region (7), respectively. GIST cases in Saudi Arabia predominantly occur in individuals over 50 years of age, with a slight male predominance (6).

## Case report

A 31-year-old Saudi male presented to the emergency department at King Khalid Hospital in Tabuk, Saudi Arabia, with central abdominal pain for four days. The pain he experienced had a sudden onset, became progressively worse, and did not radiate. He used simple analgesics to alleviate his pain, but they were not effective. His pain was associated with intermittent progressive nausea and vomiting. He did not have a bowel movement for four days. He had a subjective, intermittent fever and night sweats associated with low oral intake and dark urine. He denied loss of weight. The systemic inquiry was uneventful. He denied a history of previous surgery, blood transfusion, and trauma. An investigation into his familial background yielded little of note, except for a father with diabetes and hypertension. Interrogation of his drug and allergy history was negative. On arrival at the emergency department, physical examination concluded that he was hypotensive with his blood pressure at 96/76 mm Hg, tachycardiac (pulse rate at 110/min), and tachypnoeic with a respiratory rate of 24/min. His temperature was 39.9 degrees Celsius. His abdomen exhibited generalised rigidity and tenderness, which were most pronounced in the hypogastric area and accompanied by rebound tenderness.

**Figure 1:**
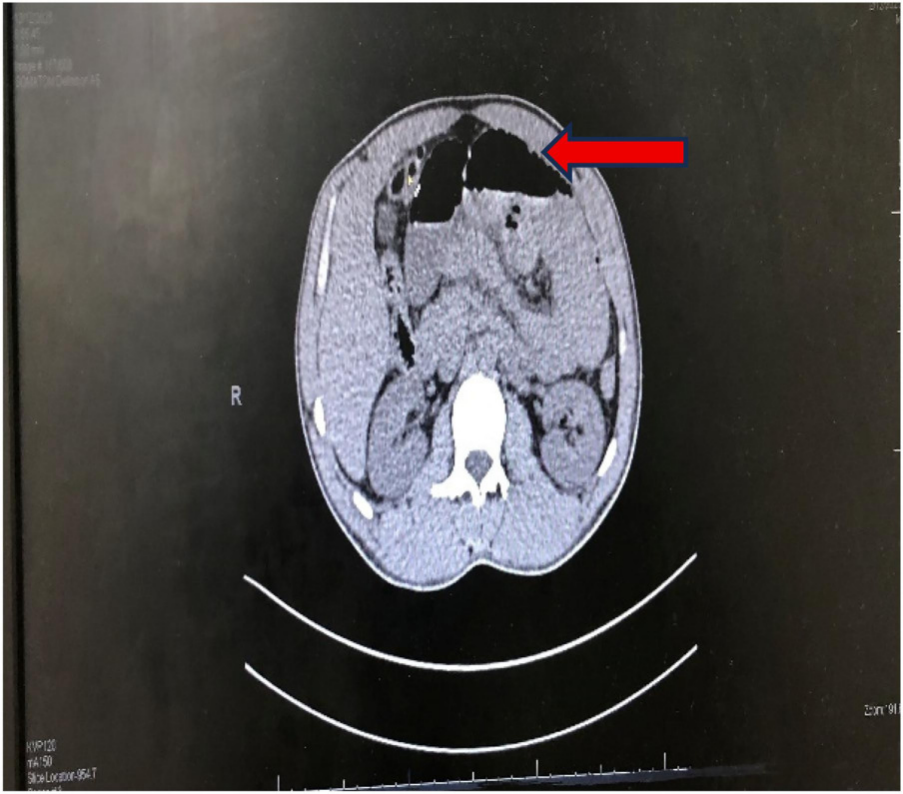
Dilated obstructed small bowel.

**Figure 2:**
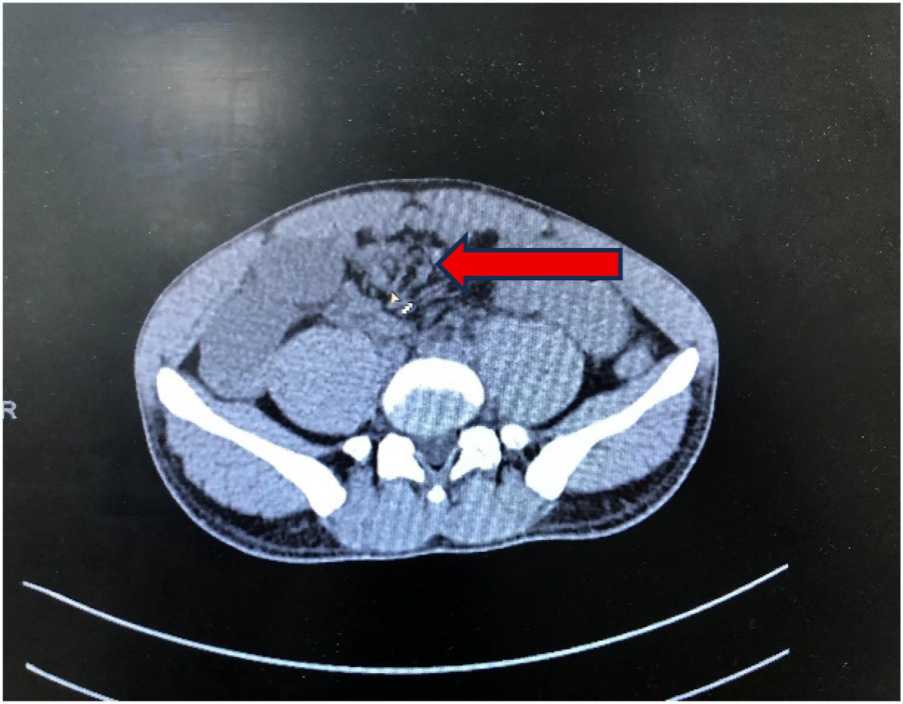
Neoplasm at distal ileum with central necrosis.

His haematological report showed leucocytosis at 26 cells/μL (per microlitre) with polymorphic neutrophilic predominance. The haemoglobin was (Hb) 15.4 grams/dL. His abdominal CT with and without contrast revealed a well-defined, iso-dense, large pelvic-abdominal soft tissue tumour to the right side of the midline, measuring 8 × 6 × 4 in its greatest dimensions, inseparable from the distal ileum, and associated with a dilated proximal small bowel. Nonetheless, the appendix was of normal quality (Figures 1, 2).

**Figure 3:**
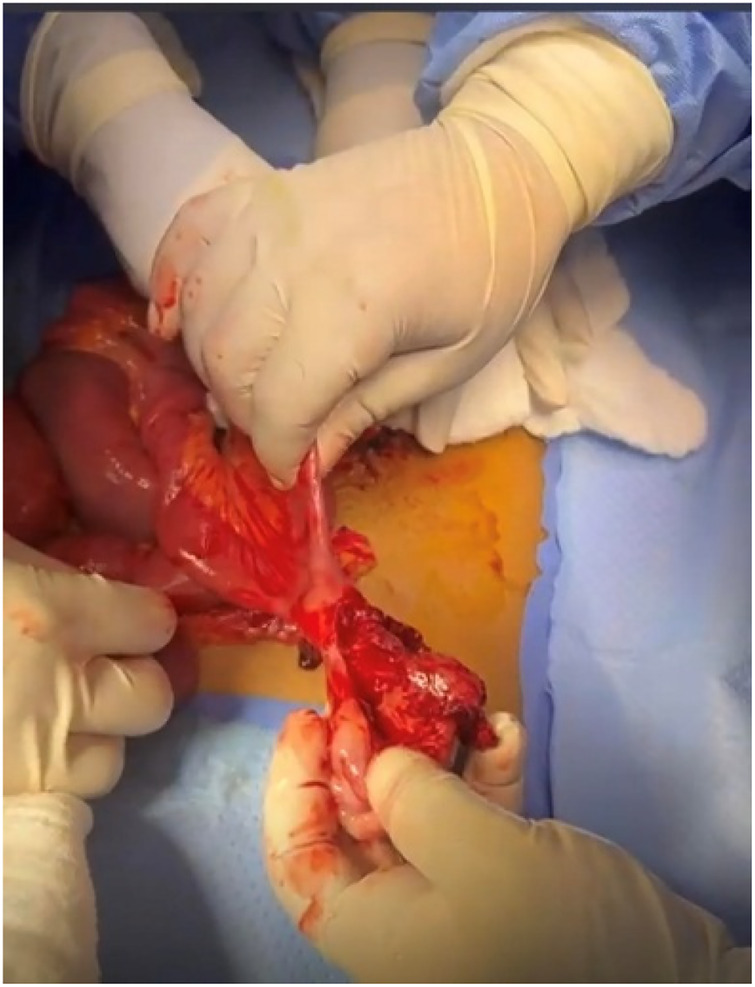
Tumour at the antimesenteric border of the distal ileum.

**Figure 4:**
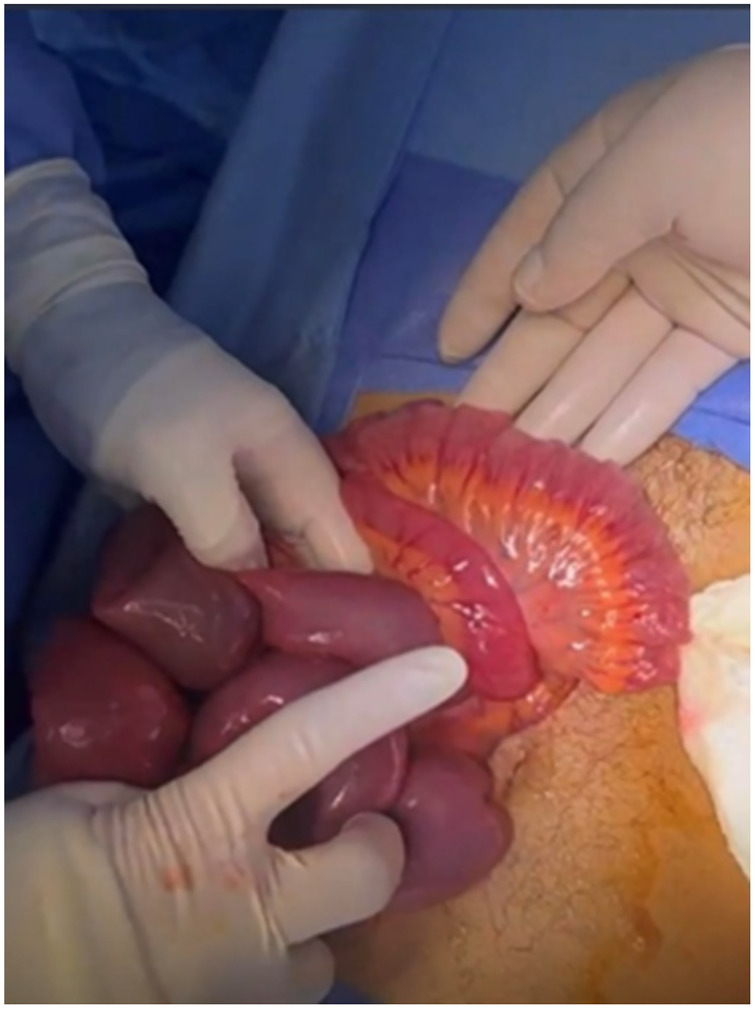
Obstructed dilated proximal small bowel.

The primary operating surgeon proceeded with an infraumbilical midline incision. The intraoperative findings demonstrated a large, heterogenous tumour at the antimesenteric border of the distal ileum with volvulus of the small bowel around the tumour (Figure 3). The proximal, small distal ileum was dusky, dilated, and hyperaemic (Figure 4). The tumour was whitish and friable, with a certain blood clot but no pus. The tumour was entangled with the obstructed distal ileum, benignly anchoring it to the pelvic peritoneum adjacent to the rectum. The primary surgeon was uncertain at that time whether the issue was a tumour or an inflammatory Meckel's diverticulum. The tumour at the antimesenteric border was resected and stapled.The remaining short, narrowed small bowel segment was resected with anti-peristaltic side-to-side anastomosis.

Macroscopic and microscopic characteristics were described in the histopathologic section. Macroscopic appearance showed three irregulars, deformed, ruptured lumps. The three lumps are 19 × 9 × 3 cm in total, and a short intestinal section was 5 cm long and 2 cm wide. We have noted smooth and inconspicuous serosa surface on a distinct intestinal segment. Intestinal mucosa was intact with luminal constriction but no gross disease. The largest tumour-sliced segment was brown and necrotic with thin whitish tissue. The solid second tumour had a yellowish sliced surface. One side of the third mass is nodular, and the other is stapled to a short bowel. Solid, white substance was noted on section 3. The 1.5 cm-wide stapled bowel segment had a patent lumen and a tiny submucosal yellow-white nodule measuring (1 × 1 cm).

Twelve tissue blocks contain representative sections: A-B section had distinct intestinal segment, Section C&D had tumour with necrosis, section E-G had a second mass, section H-L had a third mass with linked bowel. Five tissue blocks (M-Q) had extra portions. Essential processing data: Formalin-fixed, paraffin-embedded tissue blocks. Haematoxylins and Eosin (H&E) and Immunohistochemical (IHC) staining in the histological sections. Microscopic description showed an analysed sections from the three submitted masses that revealed spindle cell tumours exhibiting analogous histomorphology characteristics across all lesions. The tumour primarily consisted of spindle-shaped cells organised in crossed fascicles and whorls.

The tumour cells had elongated nuclei with vesicular to hyperchromatic chromatin and considerable eosinophilic cytoplasm. Nuclear atypia exhibited modest severity. Extensive regions of tumour necrosis were seen. A mixed mild to moderate lymphoplasmacytic infiltration was observed. Mitotic activity observed in 25 high-power fields exceeds 30 mitotic figures. No conclusive lymphovascular invasion was detected. Portions from the independently submitted segment of the small intestine exhibited intact architecture with no indication of cancer infiltration**.** Histological examination of sections from the tiny segment of colon associated with one of the masses (block K&L) demonstrated a diminutive submucosal nodule of spindle-shaped epithelioid cells with central necrosis and mixed inflammatory infiltrate, indicative of submucosal tumour involvement. The superficial mucosa was preserved.

**Figure 5:**
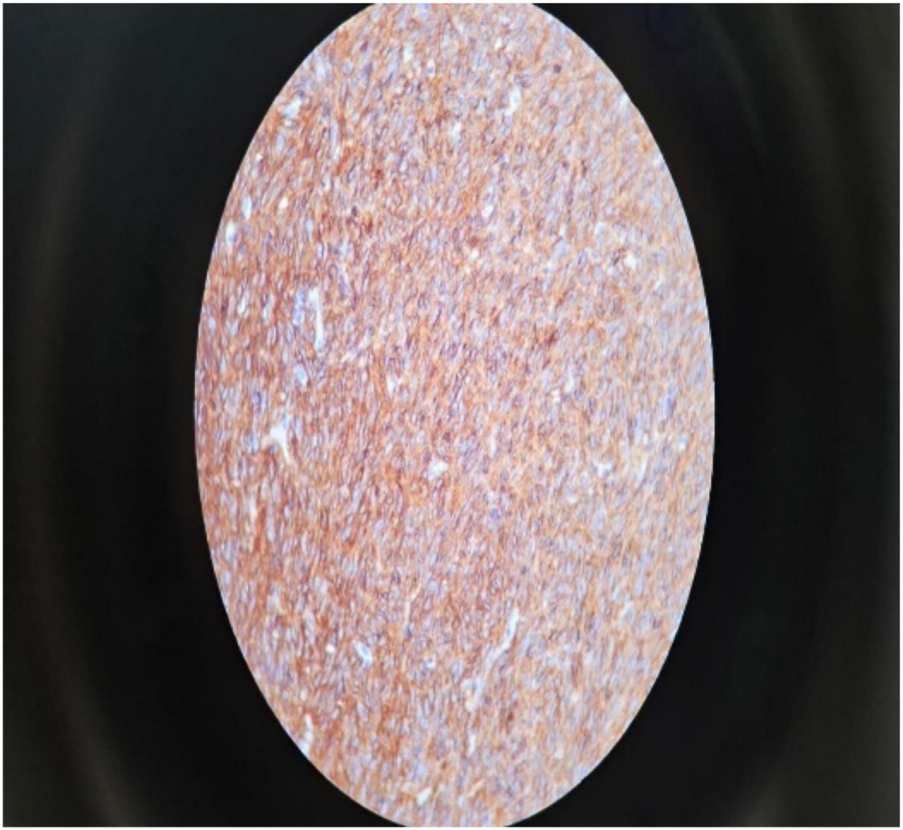
Expression of CD117.

**Figure 6:**
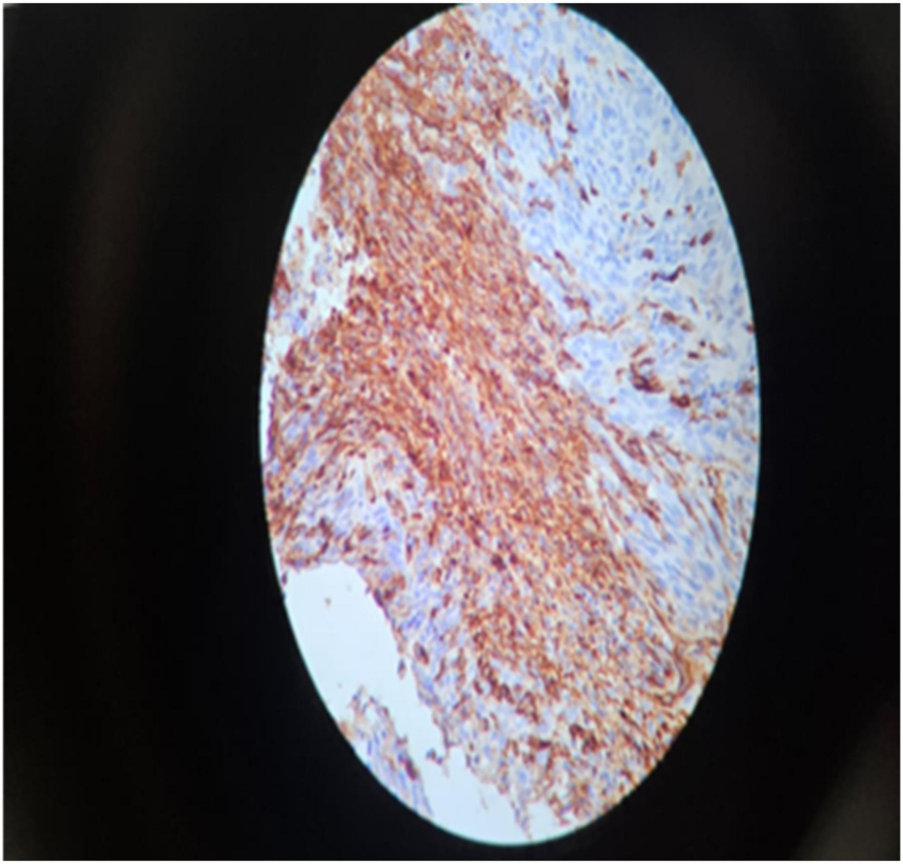
Focal patchy expression of CD34.

Immunohistochemistry analysis was performed on block E, revealing positive diffuse staining for CD117 (Figure 5) (Ckit), focal positivity for CD34 (Figure 6), and negative results for S100 and desmin. The histopathologic assessment indicated a fragmented and ruptured small intestinal tumour, with resection consistent with a high-grade spindle cell type (GIST), and no evidence of lymphovascular or perineural invasion. The tumour exhibited characteristics consistent with T4NxMx. Considering the tumour located 11 cm from the ileocecal valve and exhibiting a mitotic activity exceeding 30 mitoses per 25 high-power fields, this tumour is classified as high-risk according to the Miettinen and Lasota risk stratification guidelines. This high-risk GIST exhibits aggressive behaviour and possesses a significant potential for recurrence and metastasis. The specimen's fragmented nature and absence of clear bowel orientation hindered a reliable assessment of surgical margins.

## Discussion

Two genetic mutations are responsible for the occurrence of GIST, involving the c-kit gene, which encodes a growth factor known as stem cell factor for a transmembrane receptor. The C-KIT pathway disorder is associated with approximately 85% of GIST cases. The other genetic mutation involved is platelet-derived growth factor receptor alpha (PDGFRA-α) (6). The findings of Alghamdi HM were consistent with our study, as our immunohistochemistry analysis conducted on block E demonstrated positive diffuse staining for CD117 (Ckit), focal positivity for CD34, and negative results for S100 and desmin (6).

The differential diagnosis of GIST in the literature encompasses GI leiomyoma, GI schwannoma, sclerosing mesenteritis, small intestine benign neoplasm, gastric cancer, intestinal leiomyosarcoma, lipomas, and large intestine malignant neoplasms (8). Our study concentrated on GIST in comparison to Meckel's diverticulitis tumour, namely gastrin-producing neuroendocrine tumours (NETs) i.e., gastrinomas. The typical clinical manifestation involves a tumour that may cause intestinal obstruction, as observed in our case, or gastrointestinal tract bleeding, potentially leading to haematemesis or melena. 70% of (GIST) are symptomatic, 20% are asymptomatic, and 10% are identified post-mortem during autopsy. Even though 75% of tumours smaller than 4 centimetres present with non-specific or asymptomatic features (9–12), our patient exhibits intestinal obstruction accompanied by nausea, vomiting, constipation, and abdominal pain from a tumour measuring more than 19 × 9 × 3 in its greatest dimensions.

Various modalities, such as conventional radiography, fluoroscopy, CT scans, MRIs, and PET scans, can assess these tumours; however, a computed tomography (CT) scan plays a crucial role in the diagnosis, staging, treatment, and follow-up of these well-known GISTs, although Von Mehren et al. indicated that endoscopic ultrasound (EUS-FNA) biopsy of the tumour is superior to percutaneous biopsy due to the risks of haemorrhage and intra-abdominal tumour dissemination (13, 14), neither biopsy methods were viable in our case. DeMatteo RP et al. observed that multiple studies emphasise the importance of endoscopic ultrasound-guided fine needle aspiration, with reported accuracy ranging from 80% to 85% (15). However, this was not feasible in our case. Our patient presented acutely with a major obstruction, and the imminent risk of perforation or haemorrhage rendered these invasive biopsies inappropriate. During surgical resection, it is essential to prevent tumour rupture, as this may result in tumour spillage or recurrence. The fragility and fragmentation of the tumour during dissection precluded a favourable outcome; thus, our case necessitates rigorous surveillance with CT follow-up every 3–6 months for the next 5 years to monitor for potential recurrence, with the possible addition of Imatinib Mesylate for one or three years (16, 17). The pathological total macroscopic tumour size measures 19 × 9 × 3 cm, with mitotic activity exceeding 30 mitoses per 25 high-power fields, indicating a high risk for our patient. This aligns with the statements of Gupta P et al. “Various parameters have been evaluated as predictors of malignancy. At present, size and mitotic counts are the most effective indicators of malignant behaviour” (18). (GISTs) possess inherent malignant potential, regardless of their benign appearance. Tumours smaller than 5 cm are typically classified as low risk, whereas those larger than 5 cm are considered malignant. While a size of <5 cm is generally considered reassuring, it is not always possible to predict benignity, as there remains a potential for metastasis (19). Considering the classification of our case as high risk, we chose to collaborate with our oncologic department due to the elevated risk of metastatic recurrence associated with GISTs. Recurrence typically occurs in the liver (65%), the peritoneal surface (50%), or both (20%) (20). The response of GIST to conventional chemotherapy is minimal, at less than 10%, and radiotherapy is utilised primarily for pain relief or in instances of intraperitoneal haemorrhage (18). Imatinib mesylate is a potent inhibitor of the tyrosine kinase PDGFR and the c-kit receptor. The two-year survival rate following imatinib treatment is over 70%, with 50% of patients exhibiting no disease progression (21). The optimal dosage of imatinib has not been established; however, current data indicate no additional benefit with concentrations exceeding 400 mg/day (22). The prevalent side effects of the medication include oedema, rash, nausea, diarrhoea, myalgia, weariness, headache, and abdominal discomfort (23). Primary resistance to imatinib is infrequent, impacting about 15% of patients (24). Surgical debulking has the potential to extend survival in patients with metastatic disease, provided that the residual disease is responsive to pharmacological treatment. Due to the propensity for most GISTs to recur during the initial 3–5 years, rigorous follow-up is essential during this timeframe (25). The National Comprehensive Cancer Network recommends a contrast CT scan of the belly and pelvis every 3–6 months for 3–5 years, followed by annual scans thereafter (25). Flexible upper endoscopy is conducted at 6 months and 1 year postoperatively, followed by annual examinations for 2 years. positron emission tomography (PET)scans of the abdomen, magnetic resonance imaging (MRI), or chest CT scans are conducted if abnormalities are detected in any surveillance tests (26).

## Conclusion and recommendation

During surgical resection, it is essential to prevent tumour rupture, as this may result in tumour spillage or recurrence. The fragility and fragmentation of the tumour during dissection precluded a favourable outcome; thus, our case necessitates rigorous surveillance and CT follow-up. Effective communication between the operating surgeon and the histopathologist necessitates shared terminology for specimen labelling prior to laboratory submission. Additional detailed research studies are necessary to examine the situation in Saudi Arabia concerning epidemiological aspects, treatment protocols, and follow-up procedures. Guidelines should be established regarding the agreed protocols for managing and following up on these cases.

## Limitation

An authoritative national database registry is necessary in Saudi Arabia to document on reported cases. striving for further analysis and comprehension of the condition.

## Data Availability

The original contributions presented in the study are included in the article/Supplementary Material, further inquiries can be directed to the corresponding author.
